# Impact of diabetes mellitus on arterial stiffness in a representative sample of an urban Brazilian population

**DOI:** 10.1186/1758-5996-5-45

**Published:** 2013-08-21

**Authors:** Rafael de Oliveira Alvim, Paulo Caleb Junior Lima Santos, Mariane Manso Musso, Roberto de Sá Cunha, José Eduardo Krieger, José Geraldo Mill, Alexandre Costa Pereira

**Affiliations:** 1 Laboratory of Genetics and Molecular Cardiology, Heart Institute (InCor), University of São Paulo Medical School, Av. Dr. Enéas de Carvalho Aguiar, 44 Cerqueira César, São Paulo, SP CEP 05403-000, Brazil; 2 Department of Medicine, Juiz de Fora Federal University, Juiz de Fora, MG, Brazil; 3 Department of Physiology, Espirito Santo Federal University, Espirito Santo, ES, Brazil

**Keywords:** Arterial stiffness, Diabetes mellitus, Hypertension, Brazilian population

## Abstract

**Background:**

Independent of other cardiovascular (CV) risk factors, increased arterial stiffness has been established as a predictor of morbidity and mortality. The main aim of this study was to investigate the impact of diabetes on arterial stiffness in a representative sample of an urban Brazilian population plus Amerindians.

**Methods:**

A total of 1,415 individuals from the general population were randomly selected plus 588 Amerindians from a native community in Brazil. In addition, a sub-sample of 380 individuals from the general population had 5-year follow-up data. Pulse wave velocity (PWV) was measured with a non-invasive automatic device (Complior, Colson; Garges les Gonesses, France) and increased arterial stiffness was defined as PWV ≥ 12 m/s.

**Results:**

In the overall group, diabetic individuals had higher frequencies of increased arterial stiffness and hypertension. They also had higher values of PWV, body mass index, total cholesterol, triglycerides, systolic and diastolic blood pressures compared to non-diabetic individuals (*p* < 0.01). In an analysis stratified by hypertension, PWV values and increased arterial stiffness frequency were higher in diabetic individuals in both groups (hypertensive and non-hypertensive) (*p* < 0.05). Furthermore, higher risk for increased arterial stiffness was observed in the diabetic individuals from the overall group (OR = 2.27; CI = 1.47-3.52, *p* < 0.001) and from the hypertensive group (OR = 2.70; CI = 1.58-4.75, *p* < 0.001), adjusted for covariates. Regarding the ethnic stratification, diabetic individuals from Amerindian, White, and Mulatto (mixed-race) groups had higher PWV values and a greater frequency of increased arterial stiffness compared to non-diabetic individuals. Both diabetic and non-diabetic individuals had higher PWV values after 5 years. There was no significant difference in the 5-year PWV progression in diabetic compared to non-diabetic individuals.

**Conclusions:**

These results confirm, in a sample of Brazilian population, that the presence of diabetes is associated with increased arterial stiffness and it may contribute in part to increased cardiovascular risk in diabetic patients.

## Introduction

Increased central arterial stiffness is an important determinant of cardiovascular (CV) disease risk [1,2]. Several epidemiological studies reported that increased arterial stiffness predicts mortality and morbidity, independently of other CV risk factors [3-6]. Arterial stiffness measured through carotid-femoral pulse wave velocity (PWV), a gold standard method, has been associated with measures of subclinical CV disease [7]. Furthermore, clinical studies have shown that the arterial stiffness increases with aging or various pathological processes associated with hypertension, metabolic syndrome, chronic renal disease, and diabetes [8-12].

Diabetes mellitus is associated with increased risk for CV disease and mortality. The pathophysiological mechanism underlying these associations has not been fully elucidated. However, arterial stiffness may be one important pathway linking diabetes to increased CV risk [13,14]. Diabetes may enhance arterial stiffness through pathological changes in the vascular bed, such as reduced nitric oxide bioavailability, increased oxidative stress, chronic low-grade inflammation, increased sympathetic tone and changes in type or structure of elastin and/or collagen in the arterial wall [14-16].

Based on the fact that arterial stiffness measure is an important clinical marker for CV diseases and that diabetes is recognized as a risk factor for CV diseases, our main aim was to investigate the impact of diabetes on arterial stiffness in a representative sample of the urban Brazilian population plus Amerindians.

## Methods

### Study population

A study of risk factors for CV diseases was performed in the urban population of Vitoria, Brazil, using the WHO-MONICA project guidelines [17]. The survey was conducted with just one resident of the home selected from the age group of the study. The resident selection process was carried out by means of a randomization mechanism. The research was provided to the individual selected at each home that was then invited to participate in the study, after giving his or her consent. The selected individuals were asked to attend the Cardiovascular Investigation Clinic of the University Hospital for general, laboratory, and clinical tests to be performed on the following day. Firstly, selection of 2,268 residential homes located in Vitoria was made. Of the participants invited, 1,663 individuals (either gender, 25–64 years of age) were further evaluated and attended the clinic visit. Here, data from 1,415 participants were evaluated. After 5 years, a follow-up to perform clinical tests was done in 380 patients. We also recruited 588 Amerindians to known specific characteristic of this ethnic group, derived from two different groups (Guarani and Tupinikin) living at the Aracruz Indian Reserve, Espirito Santo State on the southeast Brazilian coast [18]. This study was approved by the ethics committee for Research on Human Subjects of the Espirito Santo Federal University and National Ethics Committee for Human Research (CONEP Register Number 4599).

### Demographic data

General data were obtained through a structured interview. Weight and height were measured according to a standard protocol, and body mass index (BMI) was calculated. Subjects of the urban population underwent ethnic classification according to a validated questionnaire for the Brazilian population, and they were classified as White, Black, or Mulatto (considered racially mixed subjects, especially between White and Black) [19]. Individuals living on the Indian reserve of Aracruz were ethnically classified as Amerindians if they reported having at least grandparents living at the reserve.

### Laboratory determinations

Blood triglycerides (TG), total cholesterol (TC), high-density lipoprotein cholesterol (HDL-C), low-density lipoprotein cholesterol (LDL-C), and fasting glucose were evaluated by standard techniques in 12-h fasting blood samples. Diabetes mellitus was diagnosed by the presence of fasting glucose ≥ 126 mg/dL and/or antidiabetic drug use [20]. Hyperlipidemia was defined as TC ≥ 240 mg/dL, LDL-C ≥ 160 mg/dL, and/or use of hypolipidemic drugs [21,22].

### Blood pressure phenotypes and PWV determinations

Blood pressure was measured while the patient was in the sitting position with the use of a standard mercury sphygmomanometer on the left arm after a 5-minute rest [18]. The first and fifth phases of Korotkoff sounds were used for systolic blood pressure (SBP) and diastolic blood pressure (DBP), respectively. The SBP and DBP were calculated from two readings with a minimal interval of 10 minutes apart. The mean blood pressure (MBP) was calculated as the mean pulse pressure added to one-third of the DBP [23]. Hypertension was defined as mean SBP ≥ 140 mmHg and/or DBP ≥ 90 mmHg and/or antihypertensive drug use [24].

Carotid-femoral PWV was analyzed with an automatic device (Complior; Colson) by an experienced observer blinded to clinical characteristics. Briefly, common carotid artery and femoral artery pressure waveforms were recorded noninvasively using a pressure-sensitive transducer (TY-306- Fukuda; Fukuda; Tokyo, Japan). The measurement of PWV was performed in one period of 10–15 seconds and the carotid-femoral distance was used for the PWV assessment. The distance between the recording sites (D) was measured, and PWV was automatically calculated as PWV = D/t, where (t) means pulse transit time. Measurements were repeated over 10 different cardiac cycles [23]. A variable for increased arterial stiffness was defined as PWV ≥ 12 m/s [25]. The validation of this automatic method and its reproducibility has been previously described [26].

### Statistical analysis

Continuous variables data are presented as mean and standard deviation and categorical variables as frequencies. χ^2^-test was performed for the comparative analysis of the frequencies of gender, hypertension, ethnicity, and increased arterial stiffness according to the presence or absence of diabetes. The Student *t* test was used to compare age, BMI, biochemical data, blood pressure phenotypes, and PWV according to diabetes. Paired samples Student *t* test for repeated measures was performed to compare the PWV values during 5-year follow-up. In addition, comparisons stratified by ethnicity and hypertension were performed. Logistic regression analyses were performed to evaluate the association of diabetes mellitus with increased arterial stiffness plus adjustment for covariates. Also, logistic models were performed to measure the interaction between diabetes mellitus and hypertension on increased arterial stiffness. PWV values were adjusted for age, gender, MBP, ethnicity, BMI, and smoking. Arterial stiffness variable was analyzed in two models using ANCOVA (model 1: adjusted for age, gender, and MBP; and model 2: adjusted for age, gender, MBP, ethnicity, BMI, smoking, and dyslipidemia). All statistical analyses were carried out using SPSS software (version 16.0, IBM, New York, NY), with the level of significance set at *p* ≤ 0.05.

## Results

### General data of the study sample

Of the 2,003 individuals, 136 (6.8%) had diabetes. The distribution of gender (*p* = 0.11) and smokers (*p* = 0.48) between diabetic and non-diabetic groups was similar. The percentage of diabetic individuals in the Amerindian group was lower than that in the White, Mulatto, and Black groups (*p* = 0.01). Hypertension (*p* < 0.001), hyperlipidemia (*p* < 0.001), and increased arterial stiffness (*p* < 0.001) frequencies were higher in diabetic compared to non-diabetic individuals (Table 1). Age (*p* < 0.001), BMI (*p* < 0.001), total cholesterol (*p* = 0.004), triglycerides (*p* < 0.001), SBP (*p* < 0.001), DBP (*p* = 0.004), and PWV (*p* < 0.001) were higher in diabetic compared to non-diabetic individuals. In addition, individuals with diabetes had lower HDL-cholesterol (*p* < 0.001) compared to non-diabetic individuals (Table 1).

**Table 1:** General characteristics, biochemical, and hemodynamic data of the general population plus Amerindian

**Variables**	**Diabetics (n136)**	**Non-diabetics (n1867)**	**p value**
Age (years)	52.7 ± 10.0	41.8 ± 12.3	<0.001
Gender, female (%)	60.3	531	011
Race/color (%)			
Amerindian	4.1	*95.9*	
White	7.1	92.9	0.01
Intermediate	8.6	91.4	
Black	6.8	93.2	
Hypertension (%)	757	354	<0.001
Increased arterial stiffness (%)	39.0	11.7	<0.001
Smokers (%)	274	236	048
Hyperlipidemia (%)	45.0	24.4	<0.001
Body mass index (Kg/m^2^)	29.6 ± 5.9	25.7 ± 4.5	<0.001
Total cholesterol (mg/dL)	212.1 ± 69.7	199.6 ± 50.0	0.004
LDL-C (mg/dL)	1326 ± 46.3	128.8 ± 44.4	0.32
HDL-C (mg/dl)	42.4 ± 10.4	46.8 ± 13.4	<0.001
Triglycerides (mg/dL)	194.1 ± 233.9	122.3 ± 101.2	<0.001
Systolic blood pressure (mmHg)	134.9 ± 213	125.3 ± 20.1	<0.001
Diastolic blood pressure (mmHg)	85.2 ± 14.0	81.6 ± 14.1	0.004
Pulse wave velocity (m/s)	11.6 ± 2.7	9.3 ± 2.1	<0.001

Continuous data are expressed as mean ± standard deviation.

HDL-C: high density lipoprotein; LDL-C: low density lipoprotein.

Biochemical data and blood pressures were adjusted for age, gender, and ethnicity.

Analysis of PWV variable is adjusted for age, gender, mean blood pressure (MBP), ethnicity, body mass index, and smoking.

Ethnicity is categorized as Amerindian, White, Mulatto (Brown or “pardo” in Portuguese, person with admixture between White and Black) and Black.

Hypertension = mean systolic blood pressure ≥ 140 mmHg and/or diastolic blood pressure ≥ 90 mmHg or use of anti-hypertension drugs.

Increased arterial stiffness frequency = pulse wave velocity (PWV) ≥ 12m/s.

Diabetes = fasting glucose ≥ 126 mg/dL and/or use of hypoglycemic drugs.

Dyslipidemia = total cholesterol ≥ 240 mg/dL, LDL-C ≥ 160 mg/dL, and/or use of hypolipidemic drugs.

Individuals who had ever smoked more than five cigarettes per day for the last year were classified as smokers.

### Analysis of the increased arterial stiffness and PWV according to diabetes and stratified by hypertension status

Of the 2,003 subjects, 767 (38.3%) had hypertension. Overall, individuals with diabetes had higher PWV than non-diabetic individuals (11.6 ± 2.7 m/s and 9.3 ± 2.1 m/s; *p* < 0.001). In addition, the frequency of increased arterial stiffness was also higher in diabetic compared to non-diabetic individuals (39.0% and 11.7%; *p* < 0.001) (Table 2). In both non-hypertensive and hypertensive groups, the diabetic individuals had higher PWV values (*p* = 0.03, *p* < 0.001; respectively) and increased arterial stiffness frequency (*p* = 0.02, *p* < 0.001; respectively) (Table 2).

**Table 2:** Analysis of the increased arterial stiffness according to diabetes mellitus of the general population plus Amerindian

	**Diabetics**	**Non-diabetics**	***p *value**
	**(n = 136)**	**(n = 1867)**	
PWN (m/s)	11.6 ± 2.2	8.5 ± 1.7	<0.001
Increased stiffness (%)	39.0	11.7	<0.001
**Models**	**OR**	**95% CI**	***p *value**
Adjusted*^¥^	2.27	1.47-3.52	<0.001
Adjusted**^¥^	2.45	1.42-3.76	<0.001
**Stratified by hypertension**			
PWN (m/s)	10.3 ± 2.2	8.5 ± 1.7	0.03
Increased stiffness (%)	12.1	3.1	0.02
**Models**	**OR**	**95%** ±	***p *value**
Adjusted*	1.65	0.53-5.16	0.39
Adjusted**	1.33	0.38-4.70	0.62
**Hypertensive**	**Diabetics**	**Non-diabetics**	***p *value**
	**(n = 103)**	**(n = 664)**	
PWN (m/s)	12.3 ± 2.7	10.9 ± 2.1	<0.001
Increased stiffness (%)	47.6	25.9	<0.001
**Models**	**OR**	**95% CI**	***p *value**
Adjusted*	2.23	1.41-3.52	0.001
Adjusted**	2.75	1.53-4.81	<0.001

Continuous data are expressed as mean ± standard deviation.

Increased arterial stiffness = pulse wave velocity (PWV) ≥ 12m/s and, this was used as dependent variable in the logistic model.

Analysis of PWV variable is adjusted for age, gender, mean blood pressure (MBP), ethnicity, body mass index (BMI), and smoking.

* Model 1: adjusted for age, gender, and MBP.

** Model 2: adjusted for age, gender, MBP, ethnicity, BMI, smoking, and dyslipidemia.

^¥^Adjusted by model 1 plus hypertension or by model 2 plus hypertension.

Hypertension = mean systolic blood pressure ≥ 140 mmHg and/or diastolic blood pressure ≥ 90 mmHg or use of anti-hypertension drugs.

Diabetes = fasting glucose ≥ 126 mg/dL and/or use of hypoglycemic drugs.

Regarding the analysis of the odds ratio of increased arterial stiffness, the individuals with diabetes in the total group had higher risk for increased arterial stiffness compared to non-diabetic individuals, even after adjustment for model 1 plus hypertension (OR 2.27, 95% CI 1.47-3.52) or for model 2 plus hypertension (OR 2.45, 95% CI 1.42-3.76) (Table 2). Furthermore, in the hypertensive group, diabetic individuals had increased risk for increased arterial stiffness compared to non-diabetic individuals in both models 1 and 2 (OR 2.23, 95% CI 1.41-3.52 and OR 2.75, 95% CI 1.53-4.81, respectively) (Table 2). However, in the non-hypertensive group, the presence of diabetes did not determine a significantly risk for increased arterial stiffness (Table 2).

**Figure 1:**
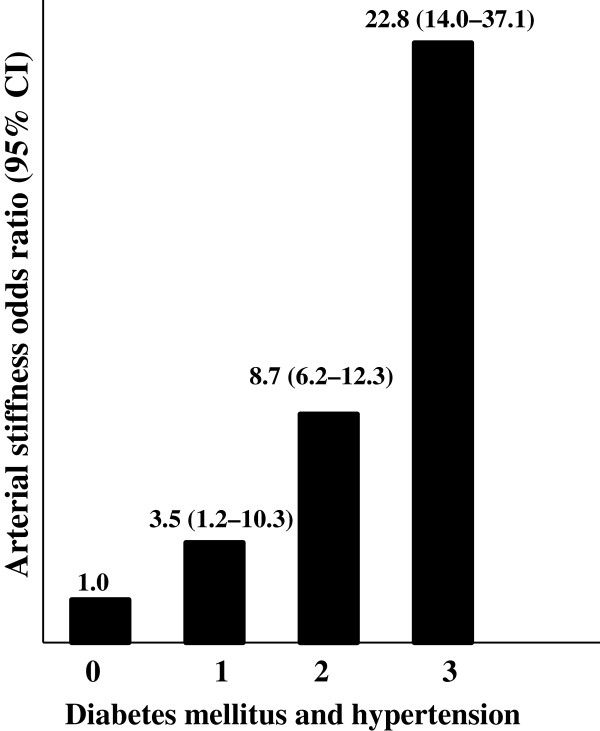
**Logistic model arterial stiffness odds ratio using diabetes mellitus and hypertension phenotypes.** Increased arterial stiffness = pulse wave velocity ≥ 12m/s. Diabetes mellitus and hypertension phenotypes are categorized as 0 (non-diabetic and non-hypertensive group), 1 (diabetic and non-hypertensive group; p = 0.02), 2 (non-diabetic and hypertensive group; p < 0.001), and 3 (diabetic and hypertensive group; p < 0.001).

In a logistic model, interaction between diabetes and hypertension on arterial stiffness was not significant (*p* = 0.62). However, a significant odds ratio was observed in patients with both phenotypes (Figure 1).

### Analysis of the increased arterial stiffness and PWV according to diabetes and stratified by ethnicity

In the study population, 588 (29.4%) were Amerindians, 533 (26.6%) Whites, 765 (38.2%) Mulatto, and 117 (5.8%) Blacks. Diabetic individuals from Amerindian, White, and Mulatto groups had higher PWV values (*p* = 0.02, *p* = 0.007, *p* < 0.001, respectively) and frequency of increased arterial stiffness (*p* < 0.001, *p* = 0.001, *p* < 0.001, respectively) compared to non-diabetic individuals (Table 3). However, in the Black group, no difference in PWV values (*p* = 0.14) and in increased arterial stiffness frequency (*p* = 0.16) was observed according to diabetes (Table 3).

**Table 3:** Analysis stratified by ethnicity for the pulse wave velocity and increased arterial stiffness according to diabetes mellitus

	**Diabetics**	**Non-diabetics**	***p *value**
**Amerindian**	**(n = 24)**	**(n = 564)**	
PWV (m/s)	11.2 ± 3.1	8.5 ± 2.0	0.02
Increased arterial stiffness (%)	33.3	10.1	<0.001
White	(11.5 ± 2.3)	9.8 ± 1.9	0.007
Increased stiffness (%)	31.6	12.3	0.001
Intermediate	(n = 66)	(n = 699)	
PWV (m/s)	11.5 ± 2.9	9.5 ±2.1	<0.001
Increased arterial stiffness (%)	45.5	11.6	<0.001
Black	(n = 8)	(n = 109)	
PWV (m/s)	12.3 ± 1.6	10.2 ± 2.5	0.14
Increased arterial stiffness (%)	37.5	17.4	0.16
PWV (m/s)	12.3 ± 1.6	10.2 ± 2.5	0.14
Increased arterial stiffness (%)	37.5	17.4	0.16

Analysis of PWV variable is adjusted for age, gender, mean blood pressure, hypertension, body mass index, and smoking.

Ethnicity is categorized as Amerindian, White, Mulatto (Brown or “*pardo*” in Portuguese, person with admixture between White and Black) and Black.

Hypertension = mean systolic blood pressure ≥ 140 mmHg and/or diastolic blood pressure ≥ 90 mmHg or use of anti-hypertension drugs.

Increased arterial stiffness = pulse wave velocity (PWV) ≥ 12m/s.

Diabetes = fasting glucose ≥ 126 mg/dL and/or use of hypoglycemic drugs.

### Analysis of the PWV values in a 5-year follow-up according to diabetes status

**Figure 2:**
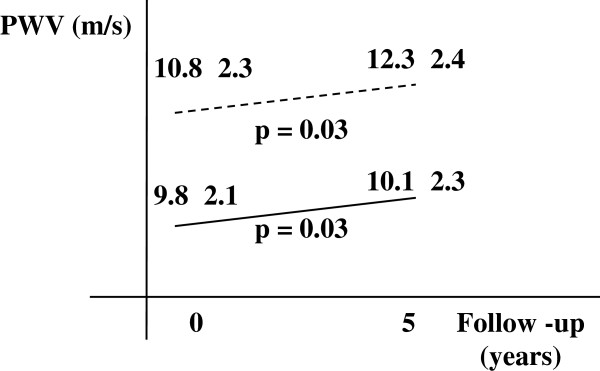
**PWV values in a 5-year follow-up to the diabetes.** The dotted line represents the individuals with diabetes (n = 25) and the solid line represents individuals without diabetes (n = 355) during the follow-up. PWV (pulse wave velocity) are expressed as mean ± standard deviation. Analysis of PWV was performed by paired samples *t* test repeated measures.

Three hundred and eighty individuals from the general population had a 5-year follow-up in which PWV values were analyzed. Regarding baseline characteristics of this subgroup, the variables gender (p = 0.15), age (p = 0.10), BMI (p = 0.22), race (p = 0.38), smoking (p = 0.55) and PWV (p = 0.99) were not different between groups. Both diabetic (n = 25) and non-diabetic (n = 355) individuals had higher PWV values after 5 years. Non-diabetic individuals increased PWV by a mean of 0.4 m/s, while diabetic individuals increased PWV by a mean of 1.5 m/s (both *p* = 0.03, performed by paired samples *t* test repeated measures). Despite the progression of values being numerically higher in the diabetic group compared with the non-diabetic group, it was not significantly different (Figure 2).

## Discussion

Our data show that diabetes status is significantly associated with PWV measurement and arterial stiffness phenotype even after adjustment for covariates. This relationship is well assessed in the literature. However, to the best of our knowledge, this is the first study to investigate the association stratified for ethnicity in an admixture population and for hypertension in individuals from an urban Brazilian population plus Amerindians.

The role of diabetes in CV risk and disease has been widely investigated. Indeed, it is generally assumed that a subject with type 2 diabetes has a similar CV risk as that of a non-diabetic individual who has sustained a myocardial infarction [27]. Our data demonstrate that subjects with diabetes had higher PWV values and higher risk for increased arterial stiffness compared to non-diabetic individuals. Corroborating our findings, Li et al. [28] reported that individuals with impaired glucose tolerance and those with diabetes had higher brachial-ankle PWV values compared to individuals with normal glucose tolerance. Galler et al. [29] identified similar findings in children and adolescents with type I diabetes compared to healthy children. Other recent studies also demonstrate the impact of diabetes on arterial stiffness [30-32]. Furthermore, an interesting study showed that individuals with diabetes with satisfactory glycemic control had higher levels of circulating endothelial progenitor cells and had lower arterial stiffness [33].

Many of the pathophysiological mechanisms responsible for vascular dysfunction in diabetes are determined by hyperglycemia, which is associated with the activation of pro-inflammatory transcription factors and increased oxidative stress, leading to vasculopathy [34]. Increased advanced glycation end-product levels may alter the important matrix of molecules in the vessel wall [35]. In addition, some studies show endothelial and vascular smooth muscle cell dysfunction in diabetic individuals compared to controls indicating that type 2 diabetes mellitus may both reduce the bioavailability of endothelial nitric oxide and attenuate sensitivity of the smooth muscle cells to nitric oxide [36-39]. All these pathways appear to be involved in mediating the hyperglycemia-associated arterial stiffness.

Diabetes and hypertension are closely associated, and it is difficult to isolate the independent and additive effects of these conditions on PWV values. Also, blood pressure measure is frequently higher in type 2 diabetes than in individuals without diabetes [40]. Moreover, analysis of the Framingham cohorts [41] showed that diabetic individuals with hypertension had higher rates of all-cause mortality and cardiovascular disease events than subjects with diabetes alone. In addition, Cardoso et al. [42] studying patients with type 2 diabetes showed that severity of hypertension, reflected by higher 24-hour pulse pressure and the greater number of anti-hypertensive drugs in use, is a risk predictor to arterial stiffness. Corroborating our findings, Tedesco et al. [43], analyzing the synergistic interaction of diabetes with hypertension on arterial stiffness, found significantly higher PWV in patients with both hypertension and diabetes compared to patients with diabetes or hypertension alone. The results from our study confirm that both diabetes and hypertension increase risk for increased arterial stiffness and that having two conditions led to even higher predicted PWV levels. In the non-hypertensive group, the absence of significant association with risk for increased arterial stiffness can be explained by the small number of diabetic individuals.

The impact of ethnic or racial differences on prevalence and morbidity of CV disease have already been reported [44,45]. Studies have shown that the prevalence of and mortality from hypertensive heart disease, stroke, and renal disease are higher among African- than Caucasian-descent individuals in the United States [44-46]. In addition, the African Americans have a higher prevalence and incidence of hypertension and end-organ damage than Whites have [47,48]. However, studies demonstrating the impact of diabetes on arterial stiffness within different ethnic groups in admixture populations are scarce. Strain et al. [49], studying Europeans and African Caribbeans, demonstrated that the diabetic individuals had higher arterial stiffness values compared to non-diabetic individuals independently of ethnicity. But, African Caribbeans with diabetes had increased stiffness compared to Europeans. Similarly to the previously mentioned comorbidities, ethnicity also is associated with arterial stiffness phenotype. A recent study from our group demonstrates that the individuals of African-descent had higher arterial stiffness and hypertension frequencies, while Amerindians had lower frequencies [23]. Here, inclusion of Amerindian individuals permitted assessment of a group with a lower frequency of hypertension beside genetic background. Our findings in an admixture population demonstrated a significant association between diabetes and increased arterial stiffness according to ethnic groups (Amerindian, White, and Mulatto), except in the Black group. Probably, this non-significant finding was the result of the small number of diabetic individuals in the Black group, but the numerical data were equally different. Amerindian group had also small number of diabetic individuals.

Regarding the analysis of the PWV values in a 5-year follow-up according to diabetes status, PWV increased 0.4 m/s in the non-diabetic group and 1.5 m/s in the diabetic group. Even these values being statistically similar - probably for the small number of diabetic patients, short-term of follow-up, and possible good treatment in the diabetic group – one can expect this increase to have significant clinical consequences. Indeed, a current study reports that an increase by a single unit (m/s) leads to a 15% higher risk of mortality from cardiovascular events [6].

Our study had some limitations. First, three measurements of office blood pressure were performed during a single study visit for hypertension classification, besides the use of antihypertensive drugs and a previous diagnosis. Also, diabetes was diagnosed by the presence of a single measurement of fasting glucose ≥126 mg/dL and/or antidiabetic drug use and a previous diagnosis. Both hypertension and diabetes diagnosis criteria could increase the likelihood of misclassification. Second, even though statistical adjustments have been made for potential confounders, in two models, residual confounding cannot be excluded. Third, our study is cross-sectional and it only provides exploratory data regarding casual relations that reaffirm the relationship between diabetes and increased arterial stiffness. We also did not collect variables, such as diabetes duration and previous renal or cardiovascular diseases. However, it may reaffirm the hypothesis that arterial stiffness acts as a trigger of CV disease in diabetic individuals. Fourth, the measurement of PWV was performed in one period of 10–15 seconds and the real carotid-femoral distance was used for the PWV assessment. These criteria differ, in part, from the new consensus [50].

## Conclusions

In conclusion, our results confirm, in a sample of Brazilian population, that the presence of diabetes is associated with increased arterial stiffness and it may contribute in part to increased cardiovascular risk in diabetic patients.

## Competing interests

The authors declare that they have no competing interests.

## Authors’ contributions

ROA and PCJLS carried out the statistical analysis and drafted the manuscript. ROA, PCJLS, MMM and ACP participated in the design of the study and manuscript preparation. RSC, JGM, JEK participated in the design of the study and were responsible for individual selection and characterization. All authors read and approved the final manuscript.
